# Fossil fuels are harming our brains: identifying key messages about the health effects of air pollution from fossil fuels

**DOI:** 10.1186/s12889-019-7373-1

**Published:** 2019-08-28

**Authors:** John Kotcher, Edward Maibach, Wen-Tsing Choi

**Affiliations:** 1 Center for Climate Change Communication, George Mason University, 4400 University Drive, MS6A8, Fairfax, VA 22030 USA; 2 Prime Group, LLC, 888 16th St NW, Washington, DC 20006 USA

**Keywords:** Air pollution, Environmental health, Public engagement, Fossil fuels, Health communication, Health education, Neurological health, Climate change

## Abstract

**Background:**

Previous research suggests that providing generalized information about the health implications of air pollution from fossil fuels may be effective at promoting public support for a transition to cleaner sources of energy. We sought to extend that work by identifying the specific messages about the health implications of air pollution from fossil fuels that are most and least concerning to people, and whether rankings of concern vary among different audiences. We also hypothesized that reading the statements would influence people’s attitudes and behavioral intentions in a manner supportive of a transition to cleaner sources of energy.

**Methods:**

We conducted a survey with a diverse sample of U.S. adults (*n* = 1644) from a non-probability internet panel. Using maximum difference scaling, participants ranked a set of ten statements that revealed which statements were the most and least concerning to them. We also measured attitudes about air pollution and energy use before and after the ranking exercise to assess changes in opinion caused by cumulative exposure to the messages.

**Results:**

Across all sub-groups examined, participants were most concerned by a message about the neurological impacts of air pollution on babies and children. After the ranking exercise, participants expressed increases in perceived health harm of air pollution and fossil fuels, a desire for more clean energy, and intention to engage in consumer advocacy to support clean energy.

**Conclusions:**

To our knowledge, this study is the first to assess how people respond to information about the neurological health harms of air pollution from fossil fuels. While efforts to communicate the cardio-pulmonary health harms of air pollution are well established, our study suggests that efforts should now be organized to communicate the neurological effects of air pollution from fossil fuels, especially the neuro-developmental effects on babies and children.

**Electronic supplementary material:**

The online version of this article (10.1186/s12889-019-7373-1) contains supplementary material, which is available to authorized users.

## Background

Modern civilization’s reliance on fossil fuels has created enormous economic progress over the past century, but it has also exacted a terrible public health toll [1–3]. Experts have long understood that air pollution from fossil fuels leads to cardio-pulmonary health harms [4], and scientists are increasingly documenting a wide array of significant health impacts associated with climate change largely driven by the use of fossil fuels [2]. More recently, neurological health harms associated with fossil fuel use are just now coming to be substantiated in the research literature [5–8]. A wide array of evidence suggests a transition to a clean energy economy would dramatically improve public health [1, 3, 9–12]. Therefore, a pressing question becomes: What is the best way to build public support for a clean energy economy? A modestly sized research literature already exists on how to communicate the cardio-pulmonary health impacts of air pollution, and there is a small but emerging research literature on communicating the health impacts of climate change. What does not yet exist at all is research examining how to communicate the neurological impacts of fossil fuels. Filling that gap is the focus of the current study.

A recent review suggests that informing people about the health implications of climate change can strengthen efforts to increase personal involvement with the issue [13]. For example, some studies have shown that a focus on the negative health effects of climate change can increase cognitive and affective engagement with the issue across political spectrum, especially among moderates and those who lean conservative [14, 15].

A separate, but related strategy is to focus on the more direct health effects of air pollution caused by burning fossil fuels as a way to increase public desire for a transition to cleaner sources of energy. Although studies in this area have primarily only tested generalized information about the health effects of fossil fuels or information about cardio-pulmonary harms, this approach shows promise for several reasons. Surveys consistently show that Americans report more concern about local air pollution than climate change [16]. People also tend to view the health effects of air pollution as closer in time and space, and more personally threatening relative to climate change [17].

Providing people with information about the health effects of air pollution from fossil fuels also seems to be effective at increasing support for clean energy policies and promoting conservation behaviors. For example, one experiment found that providing information about air pollution from fossil fuels is more effective at decreasing support for fossil fuel use and increasing support for low-carbon energy sources like solar, wind, and nuclear than information about climate change [16]. A recent field experiment found that providing parents of young children with information about the health effects of air pollution led them to have less favorable attitudes toward fossil fuels [18].

Several studies have found that focusing on the negative health effects of fossil fuels and justifying mitigation policies in terms of their health benefits was more effective at garnering support among Republicans than justifying them based upon their potential to reduce climate change [19–21]. Similarly, providing information about how pollution from coal power plants leads to premature deaths can increase support for the U.S. federal Production Tax Credit, a policy designed to promote the development of renewable energy [22]. Lastly, two field experiments have shown that information about the public health externalities of fossil fuel use, such as impacts on childhood asthma and cancer, is more effective at promoting household energy conservation than information about environmental externalities or personal cost savings associated with reduced electricity use [23, 24].

From a public health perspective, these previous studies suggest that informing people about the negative health effects of air pollution caused by fossil fuels is likely to be a useful way to increase public involvement in decisions about how best to manage those health risks. Yet, it remains unclear what specific kinds of risk information about air pollution are most engaging to people. Helping audiences understand both the severity of a threat and their susceptibility to it are two key ways to help motivate protective action [25]. Previous research suggests that public awareness of specific health problems caused by air pollution is limited, with people most commonly connecting it to asthma and other respiratory conditions, but few other specific health outcomes [26, 27]. While the contribution of air pollution to respiratory disease is well-established [4], a growing body of evidence suggests that air pollution contributes to a range of serious neurological disorders, including neurodevelopmental impacts in children and neurodegenerative effects in older adults [5–8]. Hence, a key goal of the current study was to ascertain how novel information about neurological impacts from air pollution caused by fossil fuels—and who is most vulnerable to those neurological impacts—would impact people’s attitudes toward energy use.

In the present study, we build upon previous research in two key ways. First, we sought to identify which specific messages about the health effects of air pollution from fossil fuels were the most concerning to Americans by surveying a demographically diverse group of people recruited through an online non-probability panel and asking them to rank a series of ten statements that included 1) general information about the fact that air pollution harms human health, 2) specific information about well-established health harms from air pollution caused by fossil fuels (such as respiratory and cardiovascular diseases), 3) specific information about emerging health harms from air pollution (including neurodevelopmental and neurodegenerative impacts), 4) information about which populations are the most vulnerable to health harms from air pollution (children, older adults, and low-income populations), and 5) the mechanisms by which air pollution harms human health.

In addition to identifying the most concerning messages overall, we assessed whether statement rankings differ among certain subgroups. Dual-process models of message processing stress the importance of personal relevance in shaping responses to message content [28, 29]. Because some of the statements we tested focus on the specific vulnerabilities of older adults, children, and low income populations, we suspected that members and caretakers of these groups might rank the statements differently than the general population. Additionally, political identification tends to have a major influence on attitudes toward the environment in the United States [30–32], and recent studies suggest a focus on the health effects of air pollution may be especially engaging among Republicans [19, 20]. Hence, we wanted to explore whether the messages were ranked differently among Republicans, Independents, and Democrats. This leads us to our first two research questions:

- RQ1: Which health implications of air pollution from fossil fuel use are most concerning to members of the public?
- RQ2: Do rankings of concern about the health implications of air pollution from fossil fuel use vary among different sub-groups of the public?

Beyond identifying the most concerning statements about air pollution from fossil fuels, we sought to determine whether exposure to information about the health implications of air pollution from fossil fuel use led to changes in people’s attitudes toward air pollution, fossil fuels, and clean energy. To do this, we assessed the cumulative impact of reading the ten statements by measuring participants’ attitudes both before and after the ranking task. We view this part of our study through the lens of the expectancy-value model of attitude formation, which posits that attitudes are a function of the weighted sum of evaluative beliefs held by an individual about a given object [33]. For example, one’s attitude toward fossil fuels might be based on the negative belief that they cause harm to the environment and the positive belief that are relatively inexpensive compared to other sources of energy. Depending on the relative importance that people place on harm to the environment versus economic costs, their attitude toward fossil fuels may be positive, negative, or neutral. As a result, persuasive appeals may shift people’s attitudes either by providing new considerations that expand or change the set of salient beliefs that form an attitude, or by changing the relative importance that individuals place on existing attitude-relevant beliefs. We suspect that the information about the neurological health effects of air pollution from fossil fuels will be novel and important to most individuals, adding new considerations that will influence their attitudes toward air pollution, fossil fuels, and clean energy. Because more negative attitudes may also lead people to take protective actions to reduce the threat from air pollution caused by fossil fuels [25, 34], we also assessed whether the statements increased people’s intentions to engage in consumer and political advocacy to support the use of clean energy. Given that previous studies have found messages about the health effects of air pollution to be especially persuasive among Republicans [19, 20], we were also interested to know whether changes in attitudes and behavioral intentions after the message-ranking task would be moderated by party affiliation. In particular, this partisan difference may be driven by “ceiling effects” among Democrats, such that they are already highly supportive of clean energy and thus less responsive to new information about air pollution from fossil fuels [19]. Therefore, we tested the following two hypotheses, the first of which we consider to be exploratory:

H1: Exposure to information about the health implications of air pollution caused by fossil fuels will enhance engagement in a variety of ways that favor accelerating the transition from a fossil fuel economy to a clean energy economy, including: increasing risk perceptions associated with (a) air pollution, and (b) fossil fuels; (c) increasing support for clean energy use, and (d) reducing support for fossil fuel energy use; (e) increasing opposition to fossil fuel plants near one’s home; (f) increasing support for government and industry leadership on clean energy use; and (g) increasing intention to engage in political advocacy in support of clean energy, and (h) increasing intention to engage in consumer advocacy in support of clean energy.

H2: The influence of exposure to information about the health implications of air pollution caused by fossil fuels will be moderated by party affiliation such that Republicans will demonstrate more changes in attitudes and behavioral intentions than Democrats.

## Methods

### Sample

Recruitment was conducted in December 2017 by Qualtrics, a vendor that maintains a nationwide non-probability panel of individuals who have agreed to participate in online surveys (for more information about Qualtrics, see: https://www.qualtrics.com/). Participants were a demographically diverse group of American adults (total *n* = 1644). Out of the total sample, 1025 individuals were balanced on gender, age, education, income, Hispanic ethnicity, and race to approximate the general U.S. population. The remaining 619 participants were composed of an oversample of roughly 100 additional individuals from each of the following six target groups: African American women, Hispanic women, mothers of young children, expectant mothers, childcare providers/preschool teachers, and healthcare professionals. We intentionally oversampled these groups to understand whether message rankings differed according to these individuals because they represent members and caretakers of populations that are particularly vulnerable to the health effects air pollution from fossil fuels [8, 35, 36]. Demographic characteristics of the total sample can be found in Table S1 of Additional file 1.

### Protocol

After agreeing to participate in the study, participants were asked a number of demographic questions. These questions were included at the beginning of the survey so that we could identify whether their response would fulfill outstanding demographic quotas without forcing participants to fill out the complete survey. This section was followed by questions to measure participants’ attitudes toward air pollution, fossil fuels, and clean energy, and their behavioral intentions regarding consumer and political advocacy (see below). Next, participants engaged in a maximum difference scaling exercise to elicit their ranking of ten different statements about fossil fuels and health. These ten statements were designed to present a broad range of factual information about the health consequences of air pollution caused by burning fossil fuels, including statements about well-established health harms such as asthma, cancer, and heart disease; emerging neurological health harms to children; emerging neurological health harms to older adults; mechanisms by which air pollution causes harm to health; and statements about who is most likely to be harmed by air pollution from fossil fuels (see Table 1 for exact wording of the statements). Creation of the ten statements was informed by respondent feedback from a separate study we conducted, consisting of in-depth interviews with 32 individuals. Participants in that study were provided a one-page statement about air pollution, fossil fuels, and health and then asked to answer a number of questions about their reaction to the information in the document. It is important to note that these ten statements are evidence-based, and were reviewed before use by experts on the health impacts of air pollution caused by fossil fuel use. A science synthesis document from which the statements were derived can be found here: https://bit.ly/2MTtGjO.

**Table 1:** Statement rankings including subgroup analysis of members and caretakers of vulnerable populations

Statement text	Total (*n* = 1644)	Older adult (*n* = 396)	Low income (*n* = 334)	Mother of Young Children (*n* = 465)	Expectant Mother (*n* = 150)	Childcare Provider (*n* = 201)	Healthcare Professional (*n* = 288)
Air pollution and toxic chemicals released when fossil fuels are burned can cause delays in development, reduced IQ, attention deficits, learning difficulties, behavioral problems, and autism in babies and children, even when the exposure occurs before birth	1	2	1	1	1	1	1
Air pollution caused by burning fossil fuels is causing permanent damage to the brains of many young children and older adults in America, robbing children of their full mental potential, and older adults of their mental abilities late in life.	2	1	2	2	2	2	2
New research shows that air pollution and the toxic chemicals from burning fossil fuels harms the brains of children – including babies before birth – making it more difficult for them to learn and thrive.	3	3	3	3	3	3	3
Toxic chemicals – like lead, arsenic, and mercury – that are released when coal, oil, and natural gas are burned to make energy can cause serious harm to people’s brains and mental abilities.	4	4	6	5	5	5	4
The harmful effects of air pollution from burning fossil fuels are worst for babies before and after birth, young children, the elderly, and people living or working closest to power plants and congested highways.	5	5	4	4	4	4	7
Millions of Americans suffer health problems like asthma, cancer and heart disease because of air pollution and toxic chemicals from burning fossil fuels to make energy.	6	6	5	6	7	7	6
The air pollution caused by burning fossil fuels contains tiny particles that carry toxic chemicals deep inside people’s bodies, harming their lungs, hearts and brains.	7	7	7	7	6	6	5
New research shows that the air pollution and toxic chemicals from burning fossil fuels can be especially harmful to older adults, contributing to dementia and possibly Alzheimer’s Disease.	8	8	9	10	10	8	8
Burning fossil fuels creates air pollution and releases toxic chemicals that contribute to serious – sometimes life-long – health problems for many Americans.	9	9	10	8	8	10	9
Americans living in poverty are especially at risk from air pollution and toxic chemicals that are released when fossil fuels are burned, because they often live or work close to power plants and congested highways.	10	10	8	9	9	9	10

Note: Older adult = age 55 or older; Low income = Household income less than $25,000; Mother of young children = mother with at least one child age 5 or younger

Maximum difference scaling — or MaxDiff — is a methodology that allows researchers to determine the relative preferences of respondents for a series of items [37–39]. For this study, we applied the MaxDiff technique to evaluate a series of ten statements to identify the ones that cause the most and least concern. The ten statements were shown to each respondent multiple times across eight screens, with each screen displaying a different combination of four statements. Each statement was displayed on average three times. On each screen, respondents were asked to select the statement that causes them the most concern and the one that causes them the least concern (see Additional file 1 for exact question wording). These two selections provide five data points per screen on a respondent’s preferences about the four statements displayed. For example, if statements A, B, C, and D are shown, and a respondent selects A as the greatest concern and D as the least concern, we learn that: A > B; A > C; A > D; B > D; C > D.

Through a hierarchical Bayes estimation method developed by Sawtooth Software, these data points — 40 per respondent from the eight screens — allow for the calculation of individual respondent-level utility scores for each of the items tested. The total study has 65,760 data points for the MaxDiff exercise (1644 survey interviews × 40 data points), which provide a high level of precision and confidence in the aggregated utility scores.

MaxDiff message evaluation offers distinct advantages over traditional methodologies using a Likert or numbered rating scale. The main advantage is that as a forced-choice exercise, respondents cannot rate all of the messages equally positive (or equally negative). In order to proceed through the survey they must pick and choose between the different options. The resulting data are highly differentiated and more clearly show strong and weak messages. Also, because there is no rating scale involved, MaxDiff eliminates scale-use bias, where different respondents use the same rating scale differently. For example, one respondent may only provide answers between 8 and 10 on a 0–10 scale while another respondent only provides answers between 7 and 9, despite both respondents holding similar views.

In addition to calculating the utility scores for each message, we also conducted a Total Unduplicated Reach Frequency analysis — or simply “reach analysis” — to identify the combination of messages that, taken together, are *most* highly concerning to the largest portion of respondents [40]. While the utility scores tell us the relative ranking of the messages for all respondents, a statement’s “reach” equals the percentage of respondents ranking that item as their greatest or second greatest concern. The “reach” for any two statements equals the percentage of respondents ranking either statement as their greatest or second greatest concern. Our analysis examines the total reach for every possible combination of statements and determines the package that causes the most concern.

The reach analysis provides guidance on the unique, unduplicated effect of each statement. Such an analysis would typically look for the combination of statements that greatly concerns 80% or more of the target audience, usually 3–5 statements.

After completing the MaxDiff exercise, participants were again asked the same attitude and behavioral intention questions. Lastly, participants were asked questions about their political orientation.

To examine the cumulative effect of reading and ranking the statements on participants’ attitudes and behavioral intentions, we conducted a series of mixed-design ANOVAs (analysis of variance) with time (T1- pre-test vs. T2- post-test) as a within-subjects factor and party affiliation as a between-subjects factor. A Bonferroni adjustment was used for all pairwise comparisons.

Effect size estimates for specific contrasts are provided in terms of Cohen’s *d* [41]*.* The effect size descriptors—small, medium, and large—are specific to communication research, and were derived from a quantitative review of meta-analyses [42]. A small effect refers to a Cohen’s *d* value of less than .20, a medium effect refers to values of *d* between .20 and .50, and a large effect refers to values of *d* greater than .50 [42]. A power analysis conducted with G*Power 3.1 revealed that our sample size (*n* = 1644) gave us enough power to detect a small effect size for the main effect of the within-subjects factor (*d* = 0.070) and the interaction of the within-subjects factor with partisanship (*d* = .086), assuming α = .05, β = .80 [43].

### Dependent variables

#### Perceived health risk of air pollution

Perceived risk of air pollution was measured with a single 6-point item that asked people to indicate how much of a risk they feel air pollution poses to the health of their family members. (1 = No risk at all; 2 = Very small risk; 3 = Small risk; 4 = Medium risk; 5 = Large risk; 6 = Very large risk). The question also included a “Don’t know” response option which was treated as missing data in analysis. This measure was adapted from one used in previous research [44].

#### Perceived health harm from fossil fuels

Perceived harm from fossil fuels was the average of three 5-point items that asked respondents to rate the following sources of energy in terms of how harmful they are to people’s health: *coal; oil;* and *natural gas*. The list of different energy sources assessed in the survey question also included solar, wind, hydroelectric, and geothermal. All the energy sources were presented in random order to prevent order effects. (1 = Not at all harmful; 2 = A little harmful; 3 = Moderately harmful; 4 = Very harmful; 5 = Extremely harmful). The items also included a “Don’t know” response option which was treated as missing data in analysis. Cronbach’s  α_T1_ = .73; α_T2_ = .76. This measure was adapted from one used in previous research [16, 44, 45].

#### Support for fossil fuel energy use

Support for fossil fuel energy use was the average of three 7-point items that asked participants whether they think the United States should use less, more, or about the same amount of the following sources of energy over the next several years: *coal; oil;* and *natural gas*. The list of different energy sources assessed in the survey question also included solar, wind, hydroelectric, and geothermal. All the energy sources were presented in random order to prevent order effects. (1 = Much less; 2 = Somewhat less; 3 = A little less; 4 = About the same; 5 = A little more; 6 = Somewhat more; 7 = Much more). Cronbach’s α_T1_ = .77; α_T2_ = .81. This measure was adapted from one used in previous research [16, 46].

#### Support for new fossil fuel plant near one’s home (NIMBY attitude)

Support for a new fossil fuel power plant near one’s home (a measure of “not in my back yard” or NIMBY attitude) was the average of three 7-point items that asked participants whether they would support or oppose a new power plant being built within 25 miles of their home that uses the following sources of energy: *coal; oil;* and *natural gas*. The list of different energy sources assessed in the survey question also included solar, wind, hydroelectric, and geothermal. All the energy sources were presented in random order to prevent order effects. (1 = Strongly oppose; 2 = Moderately oppose; 3 = Slightly oppose; 4 = Neither support nor oppose; 5 = Slightly support; 6 = Moderately support; 7 = Strongly support). Cronbach’s α_T1_ = .82; α_T2_ = .83. This measure was adapted from one used in previous research [16].

#### Support for clean energy use

Support for clean energy use was the average of four 7-point items that asked participants whether they think the United States should use less, more, or about the same amount of the following sources of energy over the next several years: *solar; wind; hydroelectric;* and *geothermal*. The list of different energy sources assessed in the survey question also included coal, oil, and natural gas. All the energy sources were presented in random order to prevent order effects. (1 = Much less; 2 = Somewhat less; 3 = A little less; 4 = About the same; 5 = A little more; 6 = Somewhat more; 7 = Much more). Cronbach’s α_T1_ = .83; α_T2_ = .81. This measure was adapted from one used in previous research [16, 46].

#### Support for government and industry leadership on clean energy

Support for government and industry leadership on clean energy was the average of seven 7-point items that asked whether the following should be doing more, less, or about the same amount as they are doing now to support the use of clean energy: *The President; the U.S. Congress; the U.S. Environmental Protection Agency; the U.S. Department of Energy; your state government; your local government; and corporations and industry.* All entities were presented in random order to prevent order effects. (1 = Much less; 2 = Somewhat less; 3 = A little less; 4 = About the same; 5 = A little more; 6 = Somewhat more; 7 = Much more). Cronbach’s α_T1_ = .96; α_T2_ = .96. This measure was adapted from one used in previous research to assess support for government and industry leadership on climate change [14, 46].

### Consumer advocacy intentions

Consumer advocacy intentions were the average of three 5-point items that asked how likely they are to engage in the following actions over the next 12 months to support clean energy: *contact corporate officials to urge them to support the use of clean energy; contact your local electrical utility to urge them to support the use of more clean energy; purchase clean energy from your local utility*. (1 = Not at all likely; 2 = Slightly likely; 3 = Moderately likely; 4 = Very likely; 5 = Extremely likely). Cronbach’s α_T1_ = .85; α_T2_ = .87.

### Political advocacy intentions

Political advocacy intentions were the average of three 5-point items that asked how likely they are to engage in the following actions over the next 12 months to support clean energy: *contact government officials to urge them to support the use of clean energy; join an organization working to support the use of more clean energy; vote for a political candidate because they support the use of more clean energy*. (1 = Not at all likely; 2 = Slightly likely; 3 = Moderately likely; 4 = Very likely; 5 = Extremely likely). Cronbach’s α_T1_ = .82; α_T2_ = .84. This measure was adapted from one used in previous research to assess political advocacy intentions about global warming [46].

### Moderator variable

#### Party affiliation

Party affiliation was measured with a single 7-point item that asked participants whether they think of themselves as a 1 = strong Democrat, 2 = Democrat, 3 = Independent, but lean Democrat, 4 = Independent, 5 = Independent, but lean Republication, 6 = Republican, 7 = Strong Republican.

## Results

### Statement rankings

Contrary to expectation, there was a high level of agreement among all sub-groups analyzed regarding which statements were most concerning to them. The total population sampled, as well as each sub-group isolated in the analysis (Table 1: older adults, low income, mothers of young children, expectant mothers, childcare providers, and healthcare professionals; Table 2: Democrats, Independents, Republicans) identified the same three statements as being of greatest concern. Most concerning was a statement about a range of specific neurological health problems that air pollution can cause in babies and young children. Second most concerning was a statement about the potential for air pollution to cause permanent damage to the brains of children and older adults. Third most concerning was a general statement about harm to the brains of children—including babies before birth—that makes it more difficult for them to learn and thrive.

**Table 2:** Statement rankings including subgroup analysis of Democrats, Independents, and Republicans

Statement text	Total (*n* = 1644)	Democrat (*n* = 670)	Independent (*n* = 393)	Republican (*n* = 581)
Air pollution and toxic chemicals released when fossil fuels are burned can cause delays in development, reduced IQ, attention deficits, learning difficulties, behavioral problems, and autism in babies and children, even when the exposure occurs before birth	1	1	1	1
Air pollution caused by burning fossil fuels is causing permanent damage to the brains of many young children and older adults in America, robbing children of their full mental potential, and older adults of their mental abilities late in life.	2	2	2	2
New research shows that air pollution and the toxic chemicals from burning fossil fuels harms the brains of children – including babies before birth – making it more difficult for them to learn and thrive.	3	3	3	3
Toxic chemicals – like lead, arsenic, and mercury – that are released when coal, oil, and natural gas are burned to make energy can cause serious harm to people’s brains and mental abilities.	4	5	4	4
The harmful effects of air pollution from burning fossil fuels are worst for babies before and after birth, young children, the elderly, and people living or working closest to power plants and congested highways.	5	4	7	6
Millions of Americans suffer health problems like asthma, cancer and heart disease because of air pollution and toxic chemicals from burning fossil fuels to make energy.	6	6	5	5
The air pollution caused by burning fossil fuels contains tiny particles that carry toxic chemicals deep inside people’s bodies, harming their lungs, hearts and brains.	7	7	6	7
New research shows that the air pollution and toxic chemicals from burning fossil fuels can be especially harmful to older adults, contributing to dementia and possibly Alzheimer’s Disease.	8	9	9	8
Burning fossil fuels creates air pollution and releases toxic chemicals that contribute to serious – sometimes life-long – health problems for many Americans.	9	10	8	9
Americans living in poverty are especially at risk from air pollution and toxic chemicals that are released when fossil fuels are burned, because they often live or work close to power plants and congested highways.	10	8	10	10

There were also high levels of agreement among all sub-groups regarding the three least concerning statements. The least concerning statement called attention to and explained why low-income populations are particularly vulnerable to air pollution. Second least concerning was a more general statement that invoked the “serious—sometimes life-long—health problems for many Americans” caused by air pollution. The third least concerning statement focused solely on older adults, even though it mentioned air pollution “contributing to dementia and possibly Alzheimer’s Disease” which we assumed would be conditions of grave concern to many people.

The statements ranked in the middle included a statement about the vulnerability of multiple groups (low-income populations, older adults, and children), two statements about the mechanisms by which air pollution causes health problems (one of which focused on specific toxic chemicals in air pollution, and the other focused on explaining why the “tiny particles” in air pollution are so harmful), and a statement about well-established health problems caused by air pollution (e.g., heart disease, asthma, and lung cancer).

### Reach analysis

We conducted a reach analysis to identify which four statements were ranked as the first or second most concerning by the largest number of respondents, in this case, 84%. The overall most highly ranked statement (which outlined the specific neurological health problems that air pollution can cause in children) was ranked as first or second most concerning by 40% of respondents (#1 by preference score). Next, the message that outlined some of the well-established health problems caused by air pollution was ranked as first or second most concerning by 18% of participants (#6 by preference score). The statement that emphasized the vulnerability of older adults and children to the long-lasting neurological effects of air pollution was ranked as first or second most concerning to 16% of respondents (#2 by preference score). Lastly, the message that identified the specific toxic chemicals released by burning fossil fuels that can cause neurological harm to people was ranked first or second most concerning by 10% of participants (#4 by preference score).

### Cumulative message effects

Engaging in the message-ranking exercise led to substantial (i.e., medium-sized effects) increases in participants’ perceived risk of air pollution and fossil fuels (see Table 3). Additionally, participants became less supportive of fossil fuel energy use in the United States, and more opposed to a new fossil fuel power plant being built near their home. Participants also became more likely to support clean energy use in the United States and became more supportive of government and industry efforts to embrace clean energy. Participants also became more likely to express an intention to engage in consumer advocacy, although not more likely to intend to engage in political advocacy. Taken together, these findings support our first hypothesis, with the exception of increased intention to engage in political advocacy. However, we did not find support for our second hypothesis. None of the effects of the statement-ranking exercise were moderated by partisan orientation (see Additional file 1: Tables S2-S9).

**Table 3:** Within-subjects effect of reading and ranking messages on attitudes about air pollution and energy use

	F	df	Error df	*p*-value	Effect size *d*	T1 Mean	T1 Std. Deviation	T2 Mean	T2 Std. Deviation
Perceived risk of air pollution	101.38	1	1551	< 0.001	0.27	3.82	1.53	4.22	1.49
Perceived harm of fossil fuels	96.75	1	1545	< 0.001	0.25	2.97	0.98	3.22	1.00
Desire for more fossil fuel use	25.48	1	1637	< 0.001	0.10	3.55	1.39	3.40	1.46
Desire for new fossil fuel plant near home	26.57	1	1637	< 0.001	0.09	3.45	1.55	3.31	1.56
Desire for more renewable energy use	12.71	1	1637	< 0.001	0.08	4.77	1.39	4.88	1.36
Desire for societal support for clean energy	14.52	1	1637	< 0.001	0.07	5.13	1.61	5.25	1.59
Intention to engage in consumer advocacy	5.27	1	1637	0.022	0.04	2.74	1.16	2.80	1.18
Intention to engage in political advocacy	0.06	1	1637	0.806	0.01	2.80	1.16	2.81	1.19

## Discussion

To our knowledge, our study is also the first to investigate how people respond to information about the neurological risks associated with air pollution from fossil fuels.

These results add to the growing literature on how to best communicate about the public health implications of fossil fuel use. They also extend previous research that provided an understanding of how people respond to a subtle reframing of energy and climate policy as public health issue versus an environmental, economic, or national security issue [15, 19, 20, 24].

Out of the ten statements we tested, we found that a statement about the specific neurological health threats to children caused by air pollution from fossil fuels was the most concerning message overall, and among all sub-groups except older adults (who ranked the statement second most concerning). However, this does not mean that communicators should solely focus on this one message. Our analysis suggests that in order to reach the broadest possible audience, it is also important to talk about how air pollution can have long-lasting effects on the brains of children and older adults, the toxic chemicals in air pollution that lead to neurological harm, and to address more well-established forms of health harms from air pollution such as respiratory and cardiovascular disease.

In terms of the attitudinal impact of reading the statements, we found the greatest treatment effect on risk perceptions associated with air pollution and fossil fuels, with people coming to see them as more harmful. We also observed increased support for clean energy use and decreased support for fossil fuel use. The statements were comparatively less effective at influencing people’s intentions to engage in advocacy, causing only a small increase in intention to engage in consumer advocacy and no change in intention to conduct political advocacy. This finding fits with past research which shows that messages that are effective at increasing concern and cognitive involvement with an issue do not necessarily produce a similar effect that motivates advocacy behavior [47–49]. Moreover, the statements presented to participants only included information about the health threats posed by air pollution; they did not address other factors known to predict engagement in advocacy, such as beliefs about the efficacy of solutions and protective actions [50], perceived norms about the level of activism among one’s peers [51], concerns about identifying as an activist [52], or the set of conditions that make people view civic organizations as open, friendly, and pleasant places to build social relationships while doing advocacy work [53, 54].

One interesting finding from our study is the high degree of consistency in the message rankings among the different subgroups we examined. In particular, it was remarkable that messages about neurological harm to children were ranked above the messages specifically about increased vulnerability to older adults and low-income populations, even among members of those threatened groups. This may be due to the fact that many people have a psychological predisposition to feel greater pity for children than many other social groups [55], and a desire to leave behind a positive legacy for future generations [56]. An alternative explanation is that participants in these other vulnerable groups were engaged in a form of defensive processing such that they rejected the personal relevance of the messages because they do not personally identify themselves as elderly or a member of a low-income family [57]. It is also possible that the statements about neurological harm to children were ranked highly because they were more successful at increasing perceived severity of the threat from air pollution given that they are long-lasting in nature and occurring toward the beginning of the lifespan. We did not include measures that would allow us to ascertain whether some statements caused differences in perceived severity and susceptibility among participants, but this is a promising avenue for future research that could improve our understanding of why some messages were rated as more concerning than others.

We also found little variation in statement rankings and attitudinal responses to the messages across partisan orientations. While some research has suggested that messages about air pollution may be especially effective at generating support for climate-friendly policies among Republicans, these past studies compared the effect of an air pollution message relative to one about climate change [19, 20]. Hence, it may be that there is nothing intrinsic to information about the health effects of air pollution that is especially persuasive to Republicans, but rather that information about climate change tends to be particularly unpersuasive among Republicans, contributing to partisan polarization. Furthermore, the fact that Democrats and Republicans exhibited attitude changes of similar magnitudes in response to the treatment (see Additional file 1: Tables S10-S12) suggests that ceiling effects did not influence the results, and that information about the health effects of air pollution from fossil fuels is persuasive across partisan lines.

Like any study, ours is not without limitations. First, we relied upon a quota sample drawn from non-probability internet panel for participants. While quota-matching can reduce selection bias in non-probability samples, we cannot rule out the possibility that a probability sample would yield greater external validity [58]. Our sample was also limited to U.S. adults. Given that air pollution from fossil fuels threatens global health, more research is needed to understand how people from other countries and cultures might respond to the risk information we tested. For example, intercultural differences might lead some non-U.S. groups to express greater concern about neurological harm to older adults relative to what we observed with a U.S. sample.

In the present study, we only tested ten different statements about the health effects of air pollution from fossil fuels. It is likely that even stronger and more persuasive statements about this topic could be created. Moreover, the ranking task specifically asked respondents to rank the statements in terms of how concerning they found them. It is possible that respondents may have ranked the statements differently given a different criterion, such as which message is most likely to get them to sign a petition or seek out more information about the health effects of air pollution. Future research might examine additional statements or use different criteria for ranking them.

Our assessment of the persuasive impact of the statements relied upon a within-subjects, pre-post design. By having participants answer the same set of questions twice within the same survey (before and after the ranking task) it may have sensitized some of them to the purpose of the study and artificially inflated the treatment effects we observed in the within-subjects analysis. It is also important to note here that the “treatment” in our study was the statement ranking task which likely caused participants to engage in more effortful processing of the statement content than simply reading them without ranking them. Research has shown that more effortful processing of information can lead to stronger and longer-lasting attitudinal changes [29, 59]. Thus, one interpretation of our results is that they represent a relatively high-end estimate of the persuasive impact of information about the health effects of air pollution from fossil fuels. Lastly, it is also important to note that our evaluation of the persuasive impact of the messages examined the cumulative effect of reading the entire set of ten messages, repeatedly. While the ranking task suggests which of the ten messages are likely the strongest, we cannot say how effective any individual message will be in isolation.

To address these limitations, future research might test the optimal set of messages identified in our study using a between-subjects experimental design to reduce sensitization and use a more naturalistic stimulus format such as a mock news article or action request from an advocacy organization to increase ecological validity. Partnering with civic organizations to test these messages in field experiments would also help to evaluate the ecological validity of our findings [18, 60]. Recently, social scientists have begun to investigate the key principles of effective visual communication of climate change [61]. A complementary effort should be made to assess the most effective ways to visually communicate the health effects of air pollution as a complement to textual communication.

## Conclusions

Humanity’s continued reliance on fossil fuels is contributing to one of the greatest global health threats of the twenty-first century both indirectly through climate change and directly through air pollution [3]. Our study suggests that informing people about the negative health effects of air pollution caused by burning fossil fuels can help increase public engagement around this issue. In particular, information about the neurological harm that air pollution causes to the developing brains of babies and young children seems to be especially concerning to people.

Efforts to communicate the cardio-pulmonary health harms associated with air pollution are reasonably well established (e.g., see the activities of the American Lung Association). Efforts to communicate the health harms of global warming are newly emerging (e.g., see the activities of the Medical Society Consortium on Climate and Health). Efforts should now be organized to communicate the neurotoxic harms of air pollution from fossil fuel use, especially the neurodevelopmental effects on babies and children. Such efforts have the potential to involve important new stakeholders and trusted voices that have not traditionally been involved in the campaign to clean our air and energy supplies – for the health benefit of all.

## Additional files

Additional file 1: This file contains supplementary **Tables S1–12** and question wording for the MaxDiff procedure. (DOCX 24 kb)

Additional file 2: This file contains all data underlying the analyses reported in this article. (CSV 11640 kb)

## Data Availability

All data generated or analyzed during this study are included in this published article and its supplementary information files (see Additional file 2).

## Abbreviations

ANOVA: Analysis of variance
MaxDiff: Maximum difference scaling
NIMBY: Not in my back yard
U.S.: United States

## References

[1] National Research Council. Hidden costs of energy: unpriced consequences of energy production and use. 2009.

[2] USGCRP (2016). The impacts of climate change on human health in the United States: a scientific assessment.

[3] Watts N, Amann M, Arnell N, Ayeb-Karlsson S, Belesova K, Berry H, et al. The 2018 report of the lancet countdown on health and climate change: shaping the health of nations for centuries to come. Lancet. 2018. 10.1016/S0140-6736(18)32594-7 PMC7616804 30503045

[4] Landrigan PJ, Fuller R, Acosta NJR, Adeyi O, Arnold R, Basu N (2018). The lancet commission on pollution and health. Lancet.

[5] Deborah B, Bellinger David C, Birnbaum Linda S, Conry Jeanne A, Asa B, Aimin C (2016). Project TENDR: targeting environmental neuro-developmental risks the TENDR consensus statement. Environ Health Perspect.

[6] Heusinkveld HJ, Wahle T, Campbell A, Westerink RHS, Tran L, Johnston H (2016). Neurodegenerative and neurological disorders by small inhaled particles. NeuroToxicology..

[7] WHO. Review of evidence on health aspects of air pollution: REVIHAAP project: final technical report. WHO Regional Office for Europe Copenhagen; 2013. 27195369

[8] Payne-Sturges DC, Marty MA, Perera F, Miller MD, Swanson M, Ellickson K (2019). Healthy air, healthy brains: advancing air pollution policy to protect Children’s health. Am J Public Health.

[9] Casey JA, Karasek D, Ogburn EL, Goin DE, Dang K, Braveman PA (2018). Retirements of coal and oil power plants in California: association with reduced preterm birth among populations nearby. Am J Epidemiol.

[10] Cifuentes L, Borja-Aburto VH, Gouveia N, Thurston G, Davis DL (2001). Hidden health benefits of greenhouse gas mitigation. Science..

[11] West JJ, Smith SJ, Silva RA, Naik V, Zhang Y, Adelman Z (2013). Co-benefits of mitigating global greenhouse gas emissions for future air quality and human health. Nat Clim Chang.

[12] Yang M, Chou S-Y (2018). The impact of environmental regulation on fetal health: evidence from the shutdown of a coal-fired power plant located upwind of New Jersey. J Environ Econ Manag.

[13] Hathaway J, Maibach EW. Health implications of climate change: a review of the literature about the perception of the public and health professionals. Curr Environ Health Rep. 2018:1–8. 10.1007/s40572-018-0190-3 PMC5876339 29423661

[14] Kotcher JE, Maibach E, Montoro M, Hassol SJ. How Americans respond to information about global warming health impacts: evidence from a national survey experiment. GeoHealth. 2018. 10.1029/2018GH000154 PMC7007167 32159018

[15] Myers T, Nisbet M, Maibach E, Leiserowitz A (2012). A public health frame arouses hopeful emotions about climate change. Clim Chang.

[16] Ansolabehere S, Konisky DM. Cheap and clean: how Americans think about energy in the age of global warming. MIT Press; 2014.

[17] Petrovic N, Madrigano J, Zaval L (2014). Motivating mitigation: when health matters more than climate change. Clim Chang.

[18] Hanus N, Wong-Parodi G, Hoyos L, Rauch M (2018). Framing clean energy campaigns to promote civic engagement among parents. Environ Res Lett.

[19] Feldman L, Hart PS (2018). Climate change as a polarizing cue: framing effects on public support for low-carbon energy policies. Glob Environ Change..

[20] Hart PS, Feldman L. Would it be better to not talk about climate change? The impact of climate change and air pollution frames on support for regulating power plant emissions. J Environ Psychol. 2018 Sep 12.

[21] Stokes LC, Warshaw C (2017). Renewable energy policy design and framing influence public support in the United States. Nat Energy.

[22] Goldfarb JL, Buessing M, Kriner DL (2016). Geographic proximity to coal plants and U.S. public support for extending the production tax credit. Energy Policy.

[23] Asensio O, Delmas M (2015). Nonprice incentives and energy conservation. Proc Natl Acad Sci.

[24] Asensio Omar Isaac, Delmas Magali A. (2016). The dynamics of behavior change: Evidence from energy conservation. Journal of Economic Behavior & Organization.

[25] Witte K (1992). Putting the fear back into fear appeals: the extended parallel process model. Commun Monogr.

[26] Bickerstaff K, Walker G (2001). Public understandings of air pollution: the ‘localisation’ of environmental risk. Glob Environ Change.

[27] Kotcher J, Adebayo A, Nelson A, Borth A, Maibach E, Rosenthal S (2019). Do Americans understand how air pollution from fossil fuels harms health?.

[28] Chaiken S (1980). Heuristic versus systematic information processing and the use of source versus message cues in persuasion. J Pers Soc Psychol.

[29] Petty R, Cacioppo J, Berkowitz L (1986). The elaboration likelihood model of persuasion. Advances in experimental social psychology.

[30] Dunlap RE, McCright AM, Yarosh JH (2016). The political divide on climate change: partisan polarization widens in the U.S. Environ Sci Policy Sustain Dev.

[31] Hornsey MJ, Harris EA, Bain PG, Fielding KS (2016). Meta-analyses of the determinants and outcomes of belief in climate change. Nat Clim Chang.

[32] McCright AM, Xiao C, Dunlap RE (2014). Political polarization on support for government spending on environmental protection in the USA, 1974–2012. Soc Sci Res.

[33] Ajzen I, Fishbein M (1980). Understanding attitudes and predicting social behaviour.

[34] Ajzen I (1991). The theory of planned behavior. Organ Behav Hum Decis Process.

[35] Bell ML, Ebisu K (2012). Environmental inequality in exposures to airborne particulate matter components in the United States. Environ Health Perspect.

[36] Miranda ML, Edwards SE, Keating MH, Paul CJ (2011). Making the environmental justice grade: the relative burden of air pollution exposure in the United States. Int J Environ Res Public Health.

[37] Cohen S. Maximum difference scaling: improved measures of importance and preference for segmentation. In: Sawtooth software conference proceedings, Sawtooth software, Inc. 2003. p. 61–74.

[38] Louviere JJ, Flynn TN, Marley AAJ. Best-worst scaling: theory, methods and applications: Cambridge University Press; 2015.

[39] Finn A, Louviere JJ (1992). Determining the appropriate response to evidence of public concern: the case of food safety. J Public Policy Mark.

[40] Howell J (2016). A simple introduction to TURF analysis.

[41] Cohen J (1988). Statistical power analysis for the behavioral sciences. 2nd ed.

[42] Weber R, Popova L (2012). Testing equivalence in communication research: theory and application. Commun Methods Meas.

[43] Faul F, Erdfelder E, Lang A-G, Buchner A (2007). G* power 3: a flexible statistical power analysis program for the social, behavioral, and biomedical sciences. Behav Res Methods.

[44] Akerlof K, Parker C, Winch P (2016). Public health, energy, & climate change: a Maryland statewide survey – fall 2016.

[45] Leiserowitz A, Maibach E, Rosenthal S, Kotcher J, Gustafson A, Bergquist P (2018). Energy in the American mind - December 2018.

[46] Leiserowitz A, Maibach E, Roser-Renouf C, Rosenthal S, Cutler M, Kotcher J. Politics & Global Warming, March 2018. New Haven: Yale Program on Climate Change Communication: Yale University and George Mason University; 2018.

[47] Levine AS, Kline R (2017). A new approach for evaluating climate change communication. Clim Chang.

[48] Kormos C, Gifford R (2014). The validity of self-report measures of proenvironmental behavior: a meta-analytic review. J Environ Psychol.

[49] Bergquist M, Nilsson A (2016). I saw the sign: promoting energy conservation via normative prompts. J Environ Psychol.

[50] Roser-Renouf C, Maibach EW, Leiserowitz A, Zhao X (2014). The genesis of climate change activism: from key beliefs to political action. Clim Chang.

[51] Doherty KL, Webler TN. Social norms and efficacy beliefs drive the alarmed segment’s public-sphere climate actions. Nat Clim Chang. 2016 May 16.

[52] Bashir NY, Lockwood P, Chasteen AL, Nadolny D, Noyes I (2013). The ironic impact of activists: negative stereotypes reduce social change influence. Eur J Soc Psychol.

[53] Han H (2014). How organizations develop activists: civic associations and leadership in the 21st century.

[54] Han H (2016). The organizational roots of political activism: field experiments on creating a relational context. Am Polit Sci Rev.

[55] Cuddy AJC, Fiske ST, Glick P. Warmth and competence as universal dimensions of social perception: the stereotype content model and the BIAS map. In: Advances in experimental social psychology. Academic Press; 2008. p. 61–149.

[56] Zaval L, Markowitz EM, Weber EU (2015). How will I be remembered? Conserving the environment for the sake of One’s legacy. Psychol Sci.

[57] Liberman A, Chaiken S (1992). Defensive processing of personally relevant health messages. Personal Soc Psychol Bull.

[58] Baker R, Brick JM, Bates NA, Battaglia M, Couper MP, Dever JA (2013). Summary report of the AAPOR task force on non-probability sampling. J Surv Stat Methodol.

[59] Lecheler S, de VCH (2016). How long do news framing effects last? A systematic review of longitudinal studies. Ann Int Commun Assoc.

[60] Stenhouse N (2017). Spreading success beyond the laboratory: applying the RE-AIM framework for effective environmental communication interventions at scale. Environ Commun.

[61] Wang S, Corner A, Chapman D, Markowitz E (2018). Public engagement with climate imagery in a changing digital landscape. Wiley Interdiscip Rev Clim Chang.

