# Supplementary material for: Fossil fuels are harming our brains: identifying key messages about the health effects of air pollution from fossil fuels
Source: BMC Public Health. 2019 Aug 28;19:1079. doi: 10.1186/s12889-019-7373-1 (PMC6712833; doi:10.1186/s12889-019-7373-1)
Supplement: Supplementary file 1 — This file contains supplementary Tables S1–12 and question wording for the MaxDiff procedure. (DOCX 24 kb) [file 12889_2019_7373_MOESM1_ESM.docx]

Supplementary Materials for

Fossil fuels are harming our brains: Identifying key messages about the health effects of air pollution from fossil fuels

**This file includes:**

Supplementary Tables 1-12

Question wording for MaxDiff procedure

| **Table S1. Distribution of Sample Demographics** | |
| --- | --- |
|  |  |
| Variable | Percent |
| **Gender, "What is your gender?"** |  |
| Male | 34.5 |
| Female | 65.3 |
| Other | 0.1 |
| **Age, "How old are you?"** |  |
| 18-24 | 16.3 |
| 25-34 | 26.9 |
| 35-44 | 24.2 |
| 45-54 | 8.5 |
| 55-64 | 16.8 |
| 65 and older | 7.3 |
| **Education, "What is the highest degree or level of school you have completed? If currently enrolled, mark the previous grade or highest degree received."** |  |
| Less than high school | 4.9 |
| High school graduate | 32.8 |
| Some college | 26.3 |
| Bachelor's degree | 25.9 |
| Post-graduate degree | 10.1 |
| **Income, "Please specify your household income."** |  |
| Less than $25,000 | 20.3 |
| $25,000-$49,999 | 26.1 |
| $50,000-$74,999 | 20.3 |
| $75,000-$99,999 | 11.5 |
| $100,000-$124,999 | 11.3 |
| Greater than $125,000 | 10.5 |
| **Hispanic/Latino Ethnicity, "Please specify your Hispanic/Latino ethnicity."** |  |
| Hispanic or Latino | 20.4 |
| Not Hispanic or Latino | 79.6 |
| **Race, "Please specify your race. (Check all that apply)"** |  |
| American Indian or Alaska Native | 2.3 |
| Asian | 6.8 |
| Black or African American | 17.6 |
| Native Hawaiian or Other Pacific Islander | 1.4 |
| White | 68.4 |
| Two or more races | 3.5 |
| **Party Identification, "Generally speaking, do you think of yourself as..."** |  |
| Strong Democrat | 10.3 |
| Democrat | 18.4 |
| Independent, but lean Democrat | 12.0 |
| Independent | 23.9 |
| Independent, but lean Republican | 10.9 |
| Republican | 15.2 |
| Strong Republican | 9.2 |

| **Table S2. ANOVA Summary table for perceived risk of air pollution** | | | | | | |
| --- | --- | --- | --- | --- | --- | --- |
|  | F | df | Error df | p-value | partial η2 | Effect size *d* |
| Treatment | 101.378 | 1 | 1551 | <0.001 | 0.061 | 0.27 |
| Partisanship | 9.914 | 6 | 1551 | <0.001 | 0.037 |  |
| Treatment X Partisanship | 1.12 | 6 | 1551 | 0.349 | 0.004 |  |
| *** *p*<.001, ** *p*<.01, * *p*<.05 |  |  |  |  |  |  |

| **Table S3. ANOVA Summary table for perceived harm of fossil fuels** | | | | | |  |
| --- | --- | --- | --- | --- | --- | --- |
|  | F | df | Error df | p-value | partial η2 | Effect size *d* |
| Treatment | 96.749 | 1 | 1545 | <0.001 | 0.059 | 0.25 |
| Partisanship | 8.837 | 6 | 1545 | <0.001 | 0.033 |  |
| Treatment X Partisanship | 1.341 | 6 | 1545 | 0.235 | 0.005 |  |
| *** *p*<.001, ** *p*<.01, * *p*<.05 |  |  |  |  |  |  |

| **Table S4. ANOVA Summary table for desire for more fossil fuel energy use** | | | | | |  |
| --- | --- | --- | --- | --- | --- | --- |
|  | F | df | Error df | p-value | partial η2 | Effect size *d* |
| Treatment | 25.475 | 1 | 1637 | <0.001 | 0.015 | 0.10 |
| Partisanship | 11.04 | 6 | 1637 | <0.001 | 0.039 |  |
| Treatment X Partisanship | 1.17 | 6 | 1637 | 0.321 | 0.004 |  |
| *** *p*<.001, ** *p*<.01, * *p*<.05 |  |  |  |  |  |  |

| **Table S5. ANOVA Summary table for new fossil fuel power plant near me** | | | | | |  |
| --- | --- | --- | --- | --- | --- | --- |
|  | F | df | Error df | p-value | partial η2 | Effect size *d* |
| Treatment | 26.572 | 1 | 1637 | <0.001 | 0.016 | 0.09 |
| Partisanship | 12.111 | 6 | 1637 | <0.001 | 0.043 |  |
| Treatment X Partisanship | 1.22 | 6 | 1637 | 0.294 | 0.004 |  |
| *** *p*<.001, ** *p*<.01, * *p*<.05 |  |  |  |  |  |  |

| **Table S6. ANOVA Summary table for desire for more renewable energy use** | | | | | |  |
| --- | --- | --- | --- | --- | --- | --- |
|  | F | df | Error df | p-value | partial η2 | Effect size *d* |
| Treatment | 12.713 | 1 | 1637 | <0.001 | 0.008 | 0.08 |
| Partisanship | 4.851 | 6 | 1637 | <0.001 | 0.017 |  |
| Treatment X Partisanship | 0.71 | 6 | 1637 | 0.645 | 0.003 |  |
| *** *p*<.001, ** *p*<.01, * *p*<.05 |  |  |  |  |  |  |

| **Table S7. ANOVA Summary table for desire for societal support for clean energy** | | | | | | |
| --- | --- | --- | --- | --- | --- | --- |
|  | F | df | Error df | p-value | partial η2 | Effect size *d* |
| Treatment | 14.516 | 1 | 1637 | <0.001 | 0.009 | 0.07 |
| Partisanship | 17.381 | 6 | 1637 | <0.001 | 0.06 |  |
| Treatment X Partisanship | 0.63 | 6 | 1637 | 0.71 | 0.002 |  |
| *** *p*<.001, ** *p*<.01, * *p*<.05 |  |  |  |  |  |  |

| **Table S8. ANCOVA Summary table for intention to engage in consumer advocacy** | | | | | | |
| --- | --- | --- | --- | --- | --- | --- |
|  | F | df | Error df | p-value | partial η2 | Effect size *d* |
| Treatment | 5.27 | 1 | 1637 | 0.022 | 0.003 | 0.04 |
| Partisanship | 7.415 | 6 | 1637 | <0.001 | 0.026 |  |
| Treatment X Partisanship | 0.57 | 6 | 1637 | 0.756 | 0.002 |  |
| *** *p*<.001, ** *p*<.01, * *p*<.05 |  |  |  |  |  |  |

| **Table S9. ANCOVA Summary table for intention to engage in political advocacy** | | | | | | |
| --- | --- | --- | --- | --- | --- | --- |
|  | F | df | Error df | p-value | partial η2 | Effect size *d* |
| Treatment | 0.06 | 1 | 1637 | 0.806 | 0 | 0.01 |
| Partisanship | 13.773 | 6 | 1637 | <0.001 | 0.048 |  |
| Treatment X Partisanship | 0.92 | 6 | 1637 | 0.477 | 0.003 |  |
| *** *p*<.001, ** *p*<.01, * *p*<.05 |  |  |  |  |  |  |

| **Table S10. Means and standard deviations of attitudes about air pollution and energy use among Democrats** | | | | | |
| --- | --- | --- | --- | --- | --- |
|  |  | T1 | | T2 | |
|  | Scale range | Mean | SD | Mean | SD |
| Perceived risk of air pollution | 1-6 | 4.07 | 1.51 | 4.51 | 1.39 |
| Perceived harm of fossil fuels | 1-5 | 3.14 | 0.95 | 3.38 | 0.99 |
| Desire for more fossil fuel use | 1-7 | 3.42 | 1.42 | 3.20 | 1.52 |
| Desire for new fossil fuel plant near home | 1-7 | 3.24 | 1.50 | 3.09 | 1.57 |
| Desire for more renewable energy use | 1-7 | 4.92 | 1.37 | 5.00 | 1.36 |
| Desire for societal support for clean energy | 1-7 | 5.50 | 1.53 | 5.61 | 1.49 |
| Intention to engage in consumer advocacy | 1-5 | 2.93 | 1.15 | 2.97 | 1.15 |
| Intention to engage in political advocacy | 1-5 | 3.07 | 1.11 | 3.07 | 1.11 |

| **Table S11. Means and standard deviations of attitudes about air pollution and energy use among Independents** | | | | | |
| --- | --- | --- | --- | --- | --- |
|  |  | T1 | | T2 | |
|  | Scale range | Mean | SD | Mean | SD |
| Perceived risk of air pollution | 1-6 | 3.69 | 1.55 | 4.16 | 1.53 |
| Perceived harm of fossil fuels | 1-5 | 2.90 | 0.96 | 3.19 | 1.00 |
| Desire for more fossil fuel use | 1-7 | 3.44 | 1.35 | 3.35 | 1.34 |
| Desire for new fossil fuel plant near home | 1-7 | 3.30 | 1.48 | 3.21 | 1.44 |
| Desire for more renewable energy use | 1-7 | 4.68 | 1.34 | 4.79 | 1.35 |
| Desire for societal support for clean energy | 1-7 | 5.09 | 1.62 | 5.24 | 1.60 |
| Intention to engage in consumer advocacy | 1-5 | 2.62 | 1.13 | 2.67 | 1.19 |
| Intention to engage in political advocacy | 1-5 | 2.60 | 1.13 | 2.63 | 1.23 |

| **Table S12. Means and standard deviations of attitudes about air pollution and energy use among Republicans** | | | | | |
| --- | --- | --- | --- | --- | --- |
|  |  | T1 | | T2 | |
|  | Scale range | Mean | SD | Mean | SD |
| Perceived risk of air pollution | 1-6 | 3.62 | 1.52 | 3.94 | 1.52 |
| Perceived harm of fossil fuels | 1-5 | 2.83 | 1.00 | 3.06 | 0.99 |
| Desire for more fossil fuel use | 1-7 | 3.77 | 1.37 | 3.66 | 1.41 |
| Desire for new fossil fuel plant near home | 1-7 | 3.80 | 1.59 | 3.62 | 1.58 |
| Desire for more renewable energy use | 1-7 | 4.66 | 1.42 | 4.80 | 1.37 |
| Desire for societal support for clean energy | 1-7 | 4.74 | 1.60 | 4.83 | 1.60 |
| Intention to engage in consumer advocacy | 1-5 | 2.62 | 1.17 | 2.68 | 1.18 |
| Intention to engage in political advocacy | 1-5 | 2.62 | 1.17 | 2.64 | 1.19 |

**Question wording for MaxDiff procedure**

The MaxDiff procedure begins with the following page of instructions:

On each of the next eight screens, you will find four statements about the health effects of pollution caused by burning fossil fuels to make energy. Please read each statement carefully and then select:

- the statement that causes you the most concern, and
- the statement that causes you the least concern.

The statements will repeat in different combinations across the eight screens.

On each of the following eight screens, the question format looks as follows:

In the space below, please indicate which statement causes you the MOST concern and which statement causes you the LEAST concern.

| Causes me the MOST concern (1) |  | Causes me the LEAST concern (2) |
| --- | --- | --- |
|  | Statement (1) |  |
|  | Statement (2) |  |
|  | Statement (3) |  |
|  | Statement (4) |  |
